# First-in-human phase 1 trial of the safety and immunogenicity of a recombinant adenovirus serotype 5 HVR48 (rAd5HVR48) HIV-1 vaccine

**DOI:** 10.1186/1742-4690-9-S2-O52

**Published:** 2012-09-13

**Authors:** SR Walsh, MS Seaman, JA Johnson, RP Tucker, KH Krause, M Weijtens, MG Pau, J Goudsmit, R Dolin, DH Barouch, LR Baden

**Affiliations:** 1 Beth Israel Deaconess Medical Center, Boston, MA, USA; 2 Brigham & Women's Hospital, Boston, MA, USA; 3 Crucell, Leiden, Netherlands; 4 Ragon Institute of MGH, MIT and Harvard, Boston, MA, USA

## Background

Adenovirus serotype 5 (Ad5) is a potent vector, but widespread seroprevalence may limit its potential use. Replacement of the hexon variable regions (HVR) of Ad5 with the HVR of the less prevalent Ad48 may result in a potent vector which bypasses pre-existing vector immunity.

## Methods

Recombinant Ad5 with seven HVRs derived from Ad48 and expressing the VRC EnvA test antigen (rAd5HVR48.ENVA) was made. 48 healthy volunteers who were seronegative to Ad5, Ad48, HIV-1, and HIV-2 were enrolled in a randomized, double-blind, placebo-controlled, dose-escalation phase 1 study. The first three groups of 12 subjects received doses of 10^9^, 10^10^, or 10^11^ vp of rAd5HVR48.ENVA vector (n=10/group) or placebo (n=2/group) at weeks 0, 4, and 24 and the fourth group received a single injection of 10^10^ vp or placebo. We performed pre-specified blinded immunogenicity analyses at day 56 and day 196 after the first immunization.

## Results

31/48 (65%) of subjects were female; median age at enrollment was 24 (range: 18-50). Vaccination was generally well tolerated: mild to moderate local and systemic reactogenicity was observed after the initial immunization, more commonly in the highest dose group, but typically resolved within 24h. No vaccine-associated SAEs occurred. In all four dose groups, 10 subjects per group developed positive EnvA-specific ELISA titers and EnvA-specific interferon-gamma ELISPOT responses following vaccination. Immune responses were seen two weeks following inoculation in the majority of subjects. Two subjects per group exhibited no vector- or insert-specific immune responses at any timepoint and are presumed placebo recipients.

## Conclusion

The rAd5HVR48 vector is generally safe and immunogenic in humans at all three doses. Immune responses against EnvA could be detected two weeks following the first inoculation. Ad5HVR48 is a promising new chimeric vector to evaluate novel inserts in further clinical trials.

